# Time-critical care gaps and systemic delays linked to higher mortality in severe trauma patients in Tanzania

**DOI:** 10.1186/s12873-026-01498-8

**Published:** 2026-02-10

**Authors:** Cherinet D. Osebo, Victoria J. Munthali, Laurean J. Rwanyuma, Rabi H. Ndeserua, Bryson M. Ikoshi, Respicious L. Boniface

**Affiliations:** 1 Department of Medicine, Stanford University School of Medicine, 300 Pasteur Drive, Stanford, CA 94305 USA; 2 Department of Surgery, McGill University Health Centre, Montreal, QC Canada; 3 Emergency Surgery and Obstetrics Units, Hargele Hospital, Hargele, Ethiopia; 4 Department of Orthopedics, Muhimbili Orthopedics Institute, Dar es Salaam, Tanzania; 5 Department of Anesthesiology, Muhimbili Orthopedics Institute, Dar es Salaam, Tanzania; 6 Department of Surgery, Muhimbili National Hospital, Dar es Salaam, Tanzania

**Keywords:** Trauma systems, Critical illness, Time-to-Treatment, Injury mortality, Low- and Middle-Income countries

## Abstract

**Background:**

Trauma remains a major cause of death in low-resource settings, yet prospective evidence on time-to-care, triage accuracy, and critical care allocation is scarce. We quantified care delays and predictors of mortality and evaluated whether a pragmatic, physiology- and injury-based “critical status” classification is robust and scalable.

**Methods:**

We conducted a prospective, multi-center observational study of 8,440 trauma patients presenting to emergency departments of four national referral hospitals in Tanzania (June 2023–June 2024) with 14-day follow-up. Critical status was defined by physiologic derangement—respiratory rate < 8 or > 30/min, oxygen saturation < 90%, systolic pressure < 90 mmHg, heart rate < 40 or > 130 bpm, or “Painful/Unresponsive” AVPU level—or by high-risk injury requiring urgent intervention. Patients meeting ≥ 1 criterion were classified as critical, others as non-critical. Primary outcomes were 24-hour and 14-day mortality; secondary outcomes were ICU admission and length of stay. Exposures included transfer status (direct vs. interfacility) and prehospital, triage, and definitive-care delays. Analyses used Kaplan–Meier survival, Cox regression, and multivariable logistic models (adjusted odds ratios [aOR], 95% CI).

**Results:**

Overall, 8440 traumatic patients were enrolled. Median age was 31 years (IQR 22–44); 6,393 (75.8%) were male; 5,142 (60.9%) were interfacility transfers. Overall, 3,133 (37.1%) met critical criteria and 5,307 (62.9%) were non-critical. Motor vehicle collisions caused 4,888 (57.9%) injuries. Median prehospital delay was 390 min (IQR 200–690), longer for transfers (490 vs. 320; *p* < 0.001). Overall mortality was 967(11.46%), with 255/8,440 (3.0%) occurring within the first 24 h and an additional 712/8,440 (8.4%) occurring between 24 h and 14 days, including 550/3,133 (17.6%) critical vs. 417/5,307 (7.9%) non-critical (HR 2.21, 95% CI 1.94–2.51). Critical status was independently associated with mortality (aOR 5.53, 95% CI 5.10–6.14, *p* < 0.001), and delayed definitive care was associated with higher mortality (aOR 1.35, 95% CI 1.02–1.79; *p* = 0.04). Sensitivity analyses confirmed robustness across missing-data scenarios.

**Conclusion:**

Trauma mortality in Tanzania remains high and may be avoidable with improved systems. A pragmatic physiology- and injury-based critical classification offers a feasible triage model for low-resource systems. Reducing transfer delays, strengthening ICU capacity, and expanding digital trauma registries could enable data-driven triage and may improve outcomes, supporting global surgical goals.

**Supplementary Information:**

The online version contains supplementary material available at 10.1186/s12873-026-01498-8.

## Introduction

Trauma is a leading cause of preventable death and disability worldwide, with the heaviest burden in low-resource settings, where health systems are underdeveloped, access to critical care is fragmented, and life-saving interventions are often delayed [1]. Severe trauma accounts for more years of life lost than many other conditions but receives far less attention and investment than infectious diseases [2, 3].

Timely access to effective trauma care is central to survival. Physiologic derangements such as hypotension, hypoxia, and altered consciousness are well-established predictors of early mortality if not rapidly corrected [4]. Critically injured patients, defined by reversible vital organ dysfunction or high-risk injuries requiring urgent intervention, contribute disproportionately to trauma-related deaths [5]. In high-income settings, these patients are typically managed in intensive care units; in resource-constrained environments, limited capacity often necessitates management outside of critical care areas, increasing the risk of preventable mortality [6, 7]. Recent global health policy frameworks emphasize critical care as a vital component of resilient health systems [8].

Time to definitive care is commonly evaluated using early time thresholds reported in the literature, including 60 min from injury, which provides a standardized reference for comparison across studies [9]. However, outcomes are not determined by a fixed temporal cutoff. The effect of delay depends on injury severity, physiological reserve, and system capabilities, including prehospital services, diagnostic capacity, surgical availability, and access to critical care. In well-resourced systems, severely injured patients may survive beyond early time benchmarks when advanced care is available, whereas in low-resource settings, even shorter delays may be fatal. Accordingly, time-to-care in this study is interpreted as a marker of access and system performance rather than an intrinsically protective unit of time.

Despite the importance of timely and appropriate care, systematic measurement of care intervals in low-resource settings remains uncommon. Delays may occur at multiple points along the care pathway, including prehospital transport, emergency department triage, diagnostic evaluation, operative intervention, and ICU admission, each of which has been associated with early mortality [10, 11]. Most existing studies from low-income settings are retrospective and rely on incomplete clinical records, limiting the ability to evaluate time-dependent associations with outcomes [11–13].

To address these gaps, we established a web-based trauma registry in Tanzania to prospectively collect real-time data across four national referral hospitals, capturing patient demographics, injury mechanisms, physiologic status, care timelines, and outcomes to enable analysis of system-level delays and outcomes [14, 15]. Critical status was defined pragmatically using physiologic criteria and high-risk injuries requiring urgent intervention, enabling stratified outcome analyses. Using this data, we conducted a prospective, multi-center observational study to examine associations between delays at each phase of care and 24-hour and 14-day in-hospital mortality, and to evaluate whether a physiology- and injury-based critical status classification can support risk stratification in resource-limited trauma systems.

## Materials and methods

### Study design and setting

Adhering to STROBE guidelines, between June 2023 and June 2024, we conducted a prospective observational study across four Tanzanian hospitals: Muhimbili Orthopedic Institute (MOI), the national referral center for trauma, orthopedic, and neurosurgical care, and three regional referral hospitals: Morogoro Regional Hospital, Musoma Regional Hospital, and Korogwe District Hospital. Sites were chosen to represent different geographic regions and referral levels located in distinct geographic regions of the country, collectively representing the highest levels of trauma care available in Tanzania. MOI provides continuous orthopedic and neurosurgical coverage with dedicated trauma operating capacity, whereas regional hospitals have more limited specialist availability and rely on referral pathways for definitive neurosurgical care. Across sites, the median critical care capacity per hospital was 19 intensive care unit (ICU) beds (IQR 16–24), 16 high-dependency unit (HDU) beds (IQR 12–18), and 4 emergency resuscitation bays (IQR 3–6). Hospitals were analyzed collectively to characterize national system-level care gaps rather than site-specific performance. The primary objective of this study was to identify time-critical care gaps and evaluate their association with mortality among patients with severe trauma.

### Data collection and registry process

Data were collected prospectively by trained emergency department clinicians using the Amber trauma registry, a secure, web-based, offline-capable platform accessible via standard web browsers (*Amber Collect* for bedside entry, *Amber Studio* for oversight, *Amber Server* for secure hosting; Amber Collect (cglobalsurgery.ca). Clinical data were entered at the point of care or shortly thereafter and synchronized automatically when connectivity was available. The registry captured patient demographics, injury mechanisms, physiological parameters, care timelines, interventions, and outcomes through 14 days or censored at discharge. Each patient was assigned an automated pseudonymized identifier. Registry variables were reviewed daily during hospitalization, with updates recorded as new clinical information became available. This approach minimized reliance on post-discharge abstraction while ensuring longitudinal outcome capture. The platform’s built-in automated validation rules, mandatory fields, and range checks were embedded to reduce entry errors.

### Participants and inclusion criteria

All trauma patients presenting to the emergency departments (EDs) of participating in hospitals during the study period were eligible for inclusion. Patients who were dead on arrival were excluded. There was no additional selection criterion based on age, mechanism, or injury severity. Patients were included regardless of subsequent transfer status, including those transferred out to another facility after initial stabilization.

### Exposures of interest and care delay definitions

Primary exposures of interest were *transfer status* (direct arrival vs. interfacility transfer), *injury severity* at presentation (critical vs. non-critical), and *time-to-care delays* (prehospital, triage, and definitive care). These exposures were selected based on prior trauma systems literature and operational relevance rather than biologic thresholds [16, 21–26].

#### Transfer status

Patients were categorized as direct arrival (presentation directly from the scene of injury) or interfacility transfer (initial evaluation at another facility with documented referral).

#### Critical status in trauma

Injury severity at presentation was pragmatically defined using initial physiological derangements [5, 7, 17, 18], high-risk injury patterns, or urgent intervention requirement, adapted from established trauma triage criteria and prior studies [4–7, 15, 19]. Patients were classified as *critical* if they met at least one of the following criteria, and as *non-critical* if they met none of these criteria:

1. Physiologic derangement at presentation:

- Respiratory rate < 8 or > 30 breaths/min.
- SpO₂ <90%.
- Systolic blood pressure < 90 mmHg.
- Heart rate < 40 or > 130 beats/min.
- Altered consciousness (AVPU: “P” = pain response, “U” = unresponsive).

2. High-risk injury patterns or urgent intervention needs:

- Open long-bone, pelvic, or skull fractures with hemodynamic compromise.
- Penetrating torso, head, or neck trauma.
- Any life-saving surgery within 24 h of ED arrival.

These approaches emphasized feasibility and clinical relevance in resource-limited settings [4–7, 15–19], where formal anatomic scoring systems such as the Injury Severity Score are often unavailable at the point of care [20]. The Kampala Trauma Score II (KTS II; range 0–10, with lower scores indicating greater severity) was recorded and analyzed in validated categories (≤ 6, 7–8, and 9–10). A separate polytrauma variable was not included.

#### Prehospital delay

Defined as the time from injury to arrival at the definitive care hospital. For transfer patients, this included the time to the first facility plus interfacility transfer time. When exact timestamps were unavailable, intervals were estimated from referral and ambulance records. For descriptive analyses, delays were categorized as < 60 min, 60–180 min, or > 180 min, reflecting commonly reported trauma system benchmarks rather than discrete physiologic thresholds. Given right-skewed distributions, prehospital delay was also modeled as a continuous variable in sensitivity analyses.

#### Triage delay

Defined as time from ED arrival to first clinician assessment, categorized as < 30, 30–60, or > 60 min to align with operational targets and prior studies. These cutpoints are process benchmarks rather than validated clinical thresholds, and continuous-time models were used to test robustness.

#### Definitive care delay

Defined as time from injury to initiation of definitive management, defined as emergency surgery or admission to an ICU or HDU. This measure incorporated both prehospital and in-hospital delays for direct arrivals and transfer patients. For interpretability, delays were categorized as < 60 min, 60–180 min, or > 180 min, and additionally analyzed as a continuous variable in sensitivity analyses.

***Polytrauma*** was defined as clinically significant injuries in two or more anatomical regions, with at least one potentially life-threatening injury based on clinical exam and available imaging. Assessment relied on physical exams and locally available diagnostics (X-ray, ultrasound, CT where available). Physiologic scores, including the Kampala Trauma Score, were recorded separately and not used to define polytrauma.

***Comorbidity*** was any documented pre-existing chronic condition unrelated to the index injury (e.g., hypertension, diabetes, chronic cardiac or respiratory disease), coded as present or absent.

***Preventable death*** was defined as a death potentially avoidable with timely, appropriate care given the injury and physiologic status; definitive classification requires structured expert review and cannot be determined from registry data alone [25].

### Outcomes

Primary outcomes were 24-hour and 14-day in-hospital mortality, with patients discharged alive on or before day 14 classified as survivors. No patients were discharged to hospice or designated do-not-resuscitate. Secondary outcomes included admission disposition (ward vs. ICU/HDU), length of stay (censored at discharge or 14 days), and life-saving operative procedures within 24 h of ED arrival, excluding minor bedside interventions. This framework allowed evaluation of time-critical care processes, injury severity, and short-term survival in resource-limited referral hospitals.

### Tool validation and quality assurance

Before multi-center rollout, the critical status definition was internally validated on 50 consecutive trauma patients at one national referral hospital. Three ED clinicians with standardized training independently applied the criteria at the point of care, blinded to outcomes. Inter-rater reliability was assessed against a consensus severity rating by senior clinicians and quantified using Cohen’s kappa (κ = 0.83, 95% CI 0.71–0.95), indicating excellent agreement. This confirmed that the critical status tool could be reliably applied in busy, resource-limited emergency settings. Predictive validity was further evaluated during full study implementation by examining associations between critical status and 14-day mortality using multivariable logistic regression and survival analyses, with sensitivity analyses confirming robustness across plausible data variations. The platform’s registry used predefined variables, mandatory fields, and automated validation rules to minimize entry errors. Data collectors received standardized training with periodic refreshers, and weekly audits with source verification were performed by a central data team. Missing data were assessed for randomness prior to analysis, and inter-rater consistency was periodically reassessed through independent case reviews. Continuous feedback from local investigators informed iterative database updates. All data were encrypted, pseudo-anonymized, and securely stored per institutional and ethical guidelines.

### Statistical analysis

All consecutive trauma cases were included without a prespecified sample size. Post-hoc power checks confirmed sufficient events (> 10 per covariate; Supplement 1) for regression modeling. Baseline characteristics were summarized descriptively; categorical variables were compared using chi-square tests and continuous variables using t-tests or Mann–Whitney U tests, as appropriate.

#### Regression analyses

Multivariable logistic regression examined associations between exposures (critical status, transfer status, and care delays) and binary outcomes (mortality, ICU admission, emergent surgery), reporting adjusted odds ratios (aOR) with 95% confidence intervals. Covariates were selected based on prior literature, clinical relevance, and a directed acyclic graph. Continuous variables were modeled linearly unless nonlinearity was detected, and interactions between critical status and delays were tested. Hospitals sites were included as a fixed effect, and clustered robust standard errors accounted for within-hospital correlation.

#### Survival analyses

Time-to-event outcomes were assessed using Kaplan–Meier curves and log-rank tests. Cox proportional hazards models estimated 14-day mortality hazard ratios (HR), with proportional hazards assumptions formally tested and met. Competing risks modeling was not required because 14-day outcomes were fully observed.

##### Sensitivity analyses

Of 8,440 patients, 195 (2.3%) had implausible or missing values (e.g., systolic BP of 1,100 mmHg or missing referral timestamps). After manual review, these values were treated as missing. Primary analyses used complete-case data. Sensitivity analyses modeled care delays as continuous variables and tested best- and worst-case imputation for missing critical status values (assuming missing values were non-critical or critical) to assess robustness. Data are presented as mean (SD), median (IQR), n/N (%), or OR/HR with 95% CI. Two-sided *p* < 0.05 indicated statistical significance. Analyses were performed using SAS version 9.4 (SAS Institute, Cary, NC).

## Results

### Patient characteristics

Among 8,440 trauma patients (Table 1), 3,133 (37.1%) were critically injured, while 5,307 (62.9%) were non-critical. Median age was 31 years (IQR 22–44), with most patients being male (6,393, 75.8%). The majority were transfers (5,142, 60.9%), and 5,013 (59.4%) originated from rural districts. Ambulance was the most common arrival mode (4,485, 53.1%). Motor vehicle collisions (MVCs) were the leading mechanism (4,888, 57.9%). Injury types were not mutually exclusive, and extremity injuries were most frequent (5,215, 61.8%), while head injuries accounted for 2556 (30.28%). Polytrauma was identified in 5,531 (65.5%). Fractures (any) were present in 5,101 (60.4%), of which 2,590/5101 (50.8%) were open requiring urgent surgical interventions. Median injury severity, using KTS, was 9 (IQR 8–10).

**Table 1:** Baseline characteristics of the study population across settings

Characteristic	All patients (*n* = 8,440)	Critical (*n* = 3,133)	Non-Critical (*n* = 5,307)	Died (*n* = 967)	Survived (*n* = 7473)
**Transfer Statu**s^**++**^					
Transfer-in	5,142 (60.92%)	2,349 (74.97%)	3,167 (59.68%)	623 (64.43%)	4,953 (66.28%)
Direct arrival	3,298 (39.08%)	784 (25.03%)	2,140 (40.32%)	344 (35.57%)	2,520 (33.72%)
**Age median (IQR)**	31 (22–44)	31 (23–42)	32 (19–46)	34 (21–43)	33 (25–51)
**Sex**					
Male	6,393 (75.75%)	4,215 (79.42%)	2,178 (69.52%)	733 (76.0%)	5,663 (76.0%)
Female	2,047 (24.25%)	1,092 (20.58%)	955 (30.48%)	234 (24.0%)	1,810 (24.0%)
**Trauma Origin**					
Rural	5,013 (59.39%)	1,738 (55.47%)	2,210 (41.64%)	572 (59.14%)	4,421 (59.14%)
Urban	3,427 (40.79%)	1,395 (44.52%)	3,097 (58.36%)	395 (40.86%)	3,052 (40.86%)
**Mode of Arrival**					
Ambulance	4,485 (53.14%)	1,671 (53.34%)	2,814 (53.04%)	522 (53.96%)	3,999 (53.47%)
Private vehicle	2,872 (34.05%)	1,042 (33.24%)	1,830 (34.47%)	330 (34.14%)	2,542 (34.04%)
Other	1,007 (11.94%)	420 (13.41%)	587 (11.07%)	115 (11.88%)	892 (11.93%)
**Mechanism of Injury**					
MVC	4,888 (57.91%)	1,765 (56.34%)	3,123 (58.85%)	581 (60.08%)	4,307 (57.58%)
Fall	2,800 (33.19%)	973 (31.06%)	1,827 (34.43%)	289 (29.88%)	2,511 (33.60%)
Blunt assault	309 (3.66%)	182 (5.81%)	127 (2.39%)	48 (4.96%)	261 (3.49%)
Crush	266 (3.15%)	112 (3.58%)	154 (2.90%)	37 (3.83%)	229 (3.07%)
Gunshot	102 (1.21%)	56 (1.79%)	46 (0.87%)	31 (3.21%)	71 (0.95%)
Stab	53 (0.63%)	25 (0.80%)	28 (0.53%)	7 (0.72%)	46 (0.62%)
Other	22 (0.26%)	20 (0.64%)	2 (0.04%)	9 (0.93%)	13 (0.17%)
**Injury Seriousness**					
Polytrauma	5,531 (65.53%)	1,777 (56.72%)	3,754 (70.74%)	584 (60.43%)	4,947 (66.19%)
Single injury	2,909 (34.47%)	356 (11.36%)	2,553 (48.11%)	383 (39.61%)	2,526 (33.81%)
**Injury Type**					
**Extremity** ^**$**^				255 (26.35%)	4382 (58.68%
UEF	1,456 (17.25%)	699 (22.31%)	757 (14.26%)	–	–
LEF	3,114 (36.89%)	1069 (34.12%)	2045 (37.93%	–	–
STI	553 (6.55%)	372 (4.41%)	181 (2.14%)	–	–
LED	45 (0.53%)	18 (0.57%	27 (0.51%)		
UED	67 (0.79%)	44 (1.40%)	23 (0.43%)	-	-
**Head/Neck** ^**$**^				637 (65.90%)	3067(41.06%)
Head injury	2556 (30.28%)	623 (19.89%)	1934(36.44%)		
Skull fracture	67 (0.79%)	32 (1.02%)	35 (0.66%)	–	–
Facial fracture	19 (0.23%)	9 (0.29%)	10 (0.19%)		
Neck injury	13 (0.15%)	8 (0.26%)	5 (0.09%)		
**Torso** ^**$**^				103 (10.65%)	447 (5.98%)
Spine fracture	288 (3.41%)	61 (1.95%)	227 (4.28%)	–	–
Pelvic fracture	157 (1.86%)	36 (1.15%)	121 (2.28%)	–	–
Thoracic injury	28 (0.33%)	21 (0.67%)	7 (0.13%)	–	–
Abdominal trauma	77 (0.91%)	61 (1.95%)	16 (0.30%)	–	–

MVC: motor vehicle collision. LEF: lower extremity fracture. UEF: upper extremity fracture. LED: lower extremity dislocation. UED: upper extremity dislocation. ^$^ Deaths by anatomical region. ^++^Rural refers to district-level areas; Urban refers to municipal-level areas, as defined by national administrative classifications

### Time-to-care delays

Median pre-hospital delay across all sites was 390 min (IQR 200–690), including time from injury to the first facility plus transfer for referred patients. Critically injured transferred patients experienced longer pre-hospital delays (490 min, IQR 300–720), compared to critical direct arrival (320 min, IQR 250–540; *P* < 0.001). Median triage delay was 18 min (IQR 5–35), with critically injured transferred patients triaged slightly faster (15 min, IQR 3–28) than direct arrivals. Median definitive care delay was 480 min (IQR 260–780), compared to survivors, delays were observed among the deceased (700 min, IQR 450–980; *p* < 0.00 L) (Table 2).

**Table 2:** Pre-hospital, triage, and definitive care delays by patient critical status, transfer status, and outcomes

Critical status	Transfer status	Pre-hospital delay min (IQR)	Triage delay min (IQR)	Definitive care delay min (IQR)
Critical	Direct	320 (250–540)	19 (5–36)	560 (300–770)
Critical	Transferred	490 (300–720)	15 (3–28)	550 (320–820)
**p-value**	–	< 0.001 vs. direct	< 0.001 vs. direct	0.06 vs. direct
Non-critical	Direct	300 (160–580)	21 (6–43)	580 (320–810)
Non-critical	Transferred	370 (220–620)	20 (6–40)	600 (330–830)
**p-value**	–	< 0.001 vs. direct	0.05 vs. direct	0.08 vs. direct
Death (any group)	–	610 (360–890)	35 (18–65)	700 (450–980)
Survivors (any group)	–	370 (180–660)	20 (6–40)	460 (240–750)
**p-value**	–	< 0.001 vs. survivors	< 0.001 vs. survivors	< 0.001 vs. survivors

Note: Delays are pooled across centers, as preliminary analyses showed similar distributions at all sites

### Dispositions and outcomes

Overall, in-hospital mortality was 967/8,440 (11.5%), including 550/967 (56.9%) deaths among critical patients and 417/967 (43.1%) among non-critical patients. Early mortality was 255/8,440 (3.0%) within 24 h, while 712/8,440 (8.4%) died between 24 h and 14 days.

Within the first 24 h, 3,051/8,440 (36.2%) patients had a documented disposition decision, of whom 1,787/3,051 (58.6%) were discharged directly from the ED. The remaining 5,389/8,440 (63.8%) were hospitalized and therefore at risk for outcomes beyond 24 h. Among hospitalized patients, 3,837/5,389 (71.2%) were discharged alive, and 712/5,389 (13.2%) died within 14 days. Among deaths after admission, mortality was highest in the ICU (400/712, 56.2%) followed by general wards (220/712, 30.9%). Among ICU deaths, 330/400 (82.5%) were critical, and 70/400 (17.5%) were non-critical; in wards, 99/220 (45.0%) were critical, and 121/220 (55.0%) were non-critical. These findings are descriptive and are not interpreted as causal (Table 3). Hospitals site was included as a covariate in regression models to account for clustering.

**Table 3:** Early and 14-day outcomes by disposition, care setting, and clinical presentation

Variables	All	Critical	Non-Critical	Deaths	Survived
**24-hour Disposition**	**3051**/8440(36.15%)	**1264**/3051(41.43%)	**1787**/3051(58.57%)	255/**8440** (3.0%)	—
Discharged	1787 (58.57%)	—	1781 (99.66%)	—	—
Ward admission	395 (12.95%)	122 (9.65%)	228 (12.76%)	45 (17.65%)	—
Taken to OR	359 (11.78%)	289 (22.86%)	94 (5.26%)	76 (29.80%)	—
ICU admission	246 (8.65%)	108 (8.54%)	4 (0.22%)	134 (52.55%)	—
Referred out	9 (0.11%)	6 (0.19%)	3 (0.06%)	—	—
Died	255 (8.36%)	216 (10.63%)	39 (3.83%)	—	—
**14-day Outcomes**	**5389**/8440(63.85%)	**3833**/5389(72.17%)	**1556**/5389(28.87%)	712/**8440**(8.4%)	**4677**/5389 (86.79%)
Discharged	3837 (71.20%)	673 (43.25%)	3164 (82.54%)	—	3837 (82.04%)
Hospitalized	819 (15.20%)	265 (17.03%)	554 (14.45%)	—	—
Died	712 (13.21%)	606 (38.95%)	106 (2.77%)	—	—
Referred out	21 (0.39%)	12 (0.77%)	9 (0.23%)	—	—
**Care Locations**					
General ward	2336 (43.35%)	600 (38.56%)	1736 (45.29%)	1736 (45.29%)	2116 (45.24%)
ICU	1778 (33.00%)	700 (44.99%)	1078 (28.12%)	1078 (28.12%)	1378 (29.46%)
HDU	1275 (23.65%)	256 (16.45%)	1019 (26.58%)	1019 (26.58%)	1183 (25.29%)
**Consciousness Level (AVPU) n = 8440**		*n* = 3133	*n* = 5307	*n* = 969	*n* = 6750
A – Alert	6750 (79.98%)	2000 (63.84%)	4750 (89.54%)	150 (15.52%)	6600 (88.34%)
V – Responds to verbal stimuli	1004 (11.90%)	850 (27.14%)	154 (2.90%)	350 (36.19%)	654 (8.75%)
P – Responds to painful stimuli	561 (6.65%)	561 (17.91%)	0 (0.00%)	367 (37.95%)	194 (2.60%)
U – Unresponsive	125 (1.48%)	125 (3.99%)	0 (0.00%)	81 (8.38%)	44 (0.59%)
**Vital Signs (median [IQR])**					
Heart rate (bpm)	85 (76–93)	98 (85–116)	83 (74–91)	104 (88–122)	86 (77–95)
Oxygen saturation (%)	98 (98–99)	95 (88–98)	98 (97–99)	94 (91–97)	98 (97–99)
Respiratory rate (bpm)	20 (18–22)	24 (19–32)	19 (17–21)	26 (20–34)	20 (18–22)
Systolic blood pressure*	124 (114–132)	110 (92–128)	122 (112–133)	108 (88–126)	123 (115–132)
Diastolic blood pressure	76 (68–83)	69 (58–79)	75 (67–84)	65 (55–78)	76 (69–83)
KTS**	9 (8–10)	8 (7–9)	9 (9–10)	7 (6–8)	9 (8–10)
Length of stay (days)	5 (3–9)	8 (5–14)	4 (3–7)	3 (2–5)	5 (3–8)

Notes: Denominators vary with data completeness. **KTS; Kampala Trauma Score is an injury severity score. *Blood pressure unit is mmHg

### Survival analysis

**Fig. 1:**
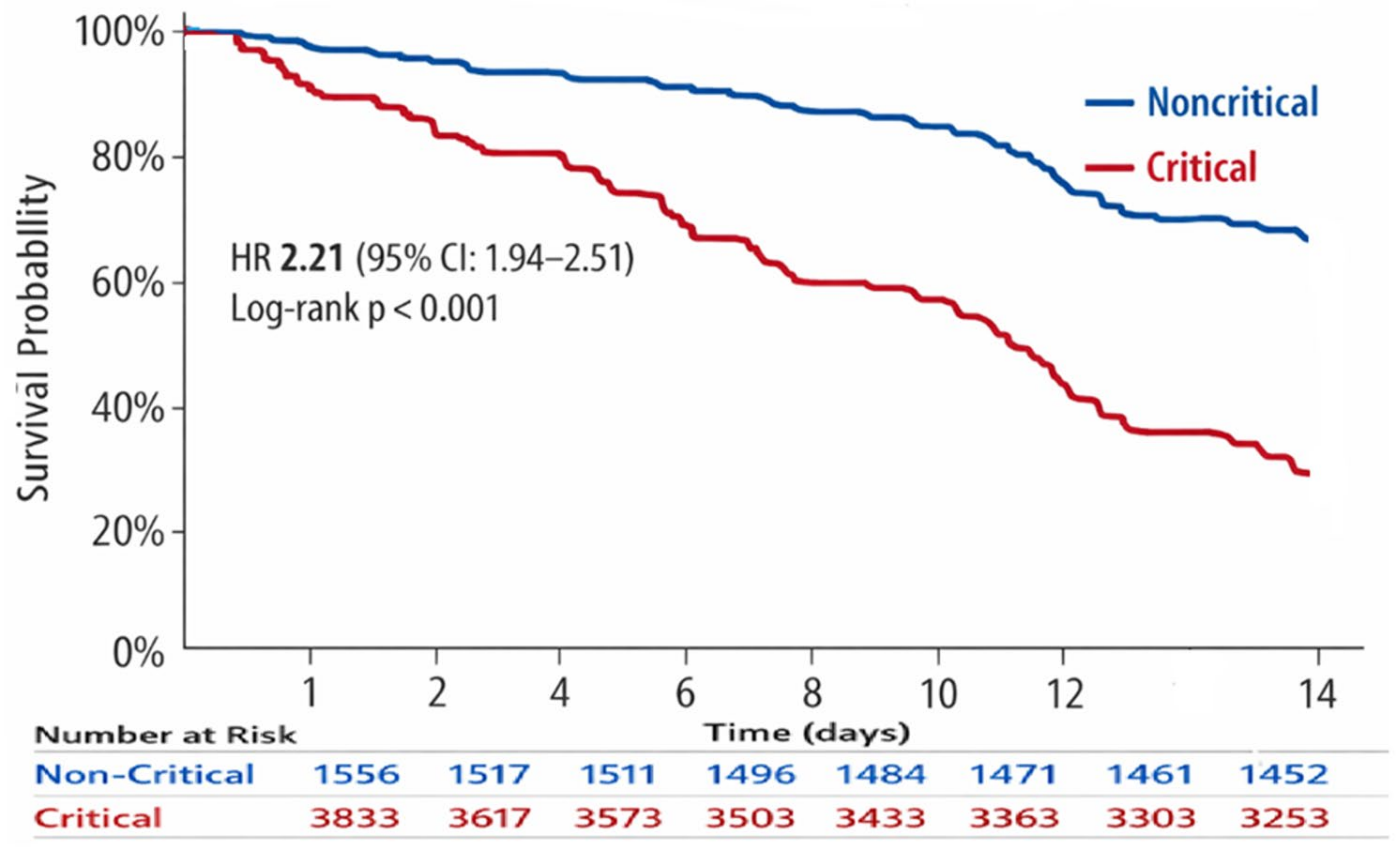
Kaplan–Meier survival curves for 14-day in-hospital mortality stratified by critical status

Figure 1 presents 14-day Kaplan–Meier survival curves for patients hospitalized beyond 24 h. Critical patients had significantly higher mortality than non-critical patients (log-rank *p* < 0.001). The hazard ratio for death in critically injured patients was 2.21 (95% CI: 1.94–2.51; *p* < 0.0001), indicating more than double the risk of death within 14 days. Patients discharged before day 14 or still hospitalized at day 14 were censored.

### Logistic regression analysis

Table 4 shows unadjusted and adjusted odds ratios for 14-day in-hospital mortality. Age was independently associated with mortality, with each 1-year increase linked to higher odds of death (adjusted OR 1.12, 95% CI 1.06–1.18; *p* < 0.001). Critical injury status was the strongest predictor of death (adjusted OR 5.53, 95% CI 5.10–6.14; *p* < 0.001). Transfer-in status was also independently associated with higher mortality (adjusted OR 1.99, 95% CI 1.73–2.32; *p* < 0.001). Head injury and spine fracture were the injury types most strongly associated with death (adjusted ORs 12.13,95% CI2.38–61.86 and 19.62, 95% CI 6.48–59.35, respectively; both *p* < 0.001). Severe injury by KTS was associated with higher mortality (adjusted OR 2.73, 95% CI 0.99–3.91; *p* = 0.05). Longer prehospital delay showed a trend toward higher mortality (adjusted OR 1.39, 95% CI 0.81–1.74; *p* = 0.19). Triage delay remained significantly associated with higher mortality after adjustment (adjusted OR 1.40, 95% CI 1.02–1.90; *p* = 0.03). Severe definitive care delay was also independently associated with higher mortality (adjusted OR 1.35, 95% CI 1.02–1.79; *p* = 0.04).

**Table 4:** Predictors of in-hospital mortality: unadjusted and adjusted logistic regression results

Variables		Unadjusted			Adjusted		
		Odds ratio	95% CI	*p*-value	Odds ratio	95% CI	*p*-value
**Age**	per 1 year	1·23	1·17–1·28	< 0·001	1.12	1.06–1.18	< 0.001
**Sex**	Female	1.14	0.78–1.04	0.90	1.291	0.99–1.68	0.06
	Male	Reference	.	.	Reference	.	.
**Injury seriousness**	Polytrauma	1.648	1.39–1.95	< 0.001	1.36	1.00-1.85	0.05
	Single injury	Reference	---	---	Reference	---	--
**Transfer status**	Transfer-in	2.20	1.93–2.53	< 0.001	1.99	1.73–2.32	< 0.001
	Direct arrival	Reference	--	--	Reference	--	--
**Care delays**							
*Prehospital*							0.01
	Moderate	1.01	0.79–1.39	0.06	1.07	0.85–1.46	0.15
	Sever	2.45	1.71–2.70	< 0.001	1.39	0.81–1.74	0.19
	Optimal	Reference	--	--	Reference	--	--
*Triage*							0.79
	Acceptable	1.08	0.96–1.22	0.21	1.04	0.89–1.22	0.59
	Delayed	1.92	0.93–2.60	0.05	1.40	1.02–1.90	0.03
	Optimal	Reference	--	--	Reference	--	--
*Definitive care*							0.51
	Moderate	0.73	0.57–0.92	0.01	1.34	0.75–2.38	0.33
	Severe	0.52	0.40–0.66	< 0.001	1.35	1.02–1.79	0.04
	Optimal	Reference	--	--	Reference	--	--
**Mechanism of injury**							0.03
	MVC	2.66	1.11–7.89	0.05	1.29	0.75–1.73	0.43
	Gunshot	3.57	1.22–11.96	0.03	1.70	0.98–2.21	0.06
	Fall	Reference	--	--	Reference	--	--
**Injury types**							< 0.001
	LEF*	3.16	1.63–5.49	0.04	2.73	1.61–4.87	0.02
	Head injury	4.13	2.04–7.48	< 0.001	12.13	2.38–61.86	< 0.001
	Spine fracture	7.89	3.15–24.06	< 0.001	19.62	6.48–59.35	< 0.001
	Thoracic injury	Reference	--	--	Reference	--	--
**Trauma origin**	Rural	1.80	0.72–1.99	0.47	2.87	1.88–3.12	0.03
	Urban	Reference	.	.	Reference	.	.
**Injury severity by KTS**							0.03
	Sever	3.79	1.65–4.96	< 0.001	2.73	1.01–3.91	0.05
	Moderate	1.85	0.65–3.06	0.29	1.49	0.68–2.64	0.34
	Mild	Reference	--	--	Reference	--	--
**Mode of arrivals**	Ambulance	2.76	2.38–3.19	< 0.001	3.21	2.22–4.64	< 0.001
	non-Ambulance	Reference	--	--	Reference	--	--
**Critical status**	Critical	5·72	5·65–6·75	< 0·001	5.53	5.10–6.14	<0.001
	Not critical	Reference	.	.	Reference	.	.

^−−^ Reference: absence of risk factor. LEF: lower extremity fracture

### Clinical predictors of mortality and sensitivity analysis

Among critical status criteria (Table 5), reduced level of consciousness was the most common (786/8,245, 9.5%) with 10.3% mortality (81/786). Injury-related criteria with the highest mortality were open fractures with hemodynamic compromise (2,950/8,245, 35.8%; 49.2% deaths). Mortality increased with the number of criteria met, from 7.4% (350/4,725) for one criterion to 75% (75/100) for more than three criteria. Overall mortality among critically injured patients was 17.6% (550/3,133). Median length of stay was 5 days (IQR 3–9), longer in critically injured patients (8 days, IQR 5–14) than non-critical patients (4 days, IQR 3–7; *p* < 0.001), and shorter for those who died (3 days, IQR 2–5) than survivors (5 days, IQR 3–8; *p* < 0.001). Critical status classification was possible in 97.7% of patients. Sensitivity analyses showed critical injury prevalence ranged from 37.1% to 39.4%, an absolute difference of 2.31% points. The direction and statistical significance of associations with mortality were consistent across all analyses.

**Table 5:** Critical predictors of mortality and sensitivity analysis of critical status across settings

Patients defined as critical by:	Severity (*n* = 8245)	Mortality		
Reduced level of consciousness (P or U)	786 (9.53%)	81/786 (10.3%)		
Hypotension (SBP < 90 mmHg)	625 (7.59%)	150/625 (24.0%)		
Extreme heart rate (HR < 40 or > 130 bpm)	500 (6.06%)	140/500 (28.0%)		
Respiratory rate (< 8 or > 30 breaths/min)	220 (2.67%)	60/220 (27.27%)		
Oxygen saturation (< 90%)	500 (6.06%)	150/500 (30.0%)		
Open fractures	2950 (35.78%)	1452/2950 (49.21%)		
Penetrating trauma to the torso/head/neck	400 (4.85%)	180/400 (45.0%)		
24-hr life-saving surgical intervention	2437 (29.59%)	76/2437 (3.12%)		
24-hr admission to ICU/HDU	246 (2.98%)	134/246 (54.47%)		
Meet one criterion	4725 (57.34%)	350/4725 (7.4%)		
Meet two criteria	1200 (14.56%)	300/1200 (25.0%)		
More than three criteria	100 (1.21%)	75/100 (75.0%)		
Mortality (whole cohort)	--	967/8440 (111.74%)		
Mortality (critical)	--	550/3133 (17.6%)		
Mortality (non-critical)	--	417/5112 (8.16%)		

Sensitivity analysis			Missing value (195/8440 (2.31%)	
Scenario	Critical	Mortality	Non-Critical	Mortality
**Base case**	3133/8245(37.99%)	550/3133(17.56%)	5112/8245(62.01%)	417/5,112(8.16%)
**Best case**: *missing data*,* considered non-critical and survived*	3133/8440(37.12%)	550/3133(17.56%)	5307/8440(62.88%)	417/5,307(7.86%)
**Worst case**: *missing data*,* considered critical and died*	3328/8440(39.43%)	745/967(77.04%)	5112/8440(60.57%)	222/5,112(4.34%)

** Clinical predictors of mortality are shown for patients with complete critical status data (*n* = 8,245), and denominators vary for mortality. Sensitivity analysis (*n* = 8,440) confirms the robustness of critical classification and associated mortality when missing values are considered in best- and worst-case scenarios. *AVPU: A – Alert, V – Responds to verbal stimuli, P – Responds to painful stimuli, U – Unresponsive

## Discussion

This study highlights the high trauma burden in a low-resource setting and exposes critical prehospital and in-hospital delays, particularly among critically injured and transferred patients, which are associated with increased mortality and signal the need for system-level interventions. The cohort, predominantly young males with extremity fractures from motor vehicle collisions, reflects global trauma patterns [27]. Over 60% were referred from peripheral facilities, and more than half originated from rural districts, emphasizing persistent barriers to timely definitive care that are independently associated with increased trauma mortality [16, 21, 27].

Across hospitals, critically injured patients, defined using physiology- and injury-based criteria, experienced longer prehospital delays for transfer-ins compared with direct arrivals, although incomplete inter-facility documentation may affect the precision of these estimates [12, 16, 21]. While causality cannot be definitively established, the association between prolonged time to definitive care and mortality suggests that system-level delays likely contributed to poor outcomes, in addition to higher baseline injury severity. This pattern is consistent with prior studies from resource-limited trauma systems where delayed recognition, stabilization, and transfer increase mortality risk [9, 22, 23].

In well-coordinated trauma systems, rapid definitive interventions such as hemorrhage control and timely surgical fixation improve survival [15, 22, 25]. Our findings indicate that longer delays to definitive care were associated with death, reflecting inefficiencies in emergency department workflows, imaging access, and surgical team mobilization. These findings support the need for structured trauma protocols and coordinated trauma response systems rather than isolated workflow improvements [22, 23, 25–27]. The high proportion of open fractures (50.8%) underscores the importance of continuous orthopedic coverage, timely access to surgical instrumentation, and infection control. In resource-limited settings, delayed or conservative fracture management increases complications, including infection and limb loss [19, 23, 25, 28, 29].

Regarding patient disposition, over 70% of patients were discharged within 14 days, yet overall mortality remained high at 11.5%, highlighting gaps in timely care delivery and escalation. Early mortality was substantial, with 3.0% of patients dying within 24 h, showcasing the immediate lethality from uncontrolled hemorrhage or severe traumatic brain injury. Mortality continued beyond the first day, with 8.4% patients dying between 24 h and 14 days, indicating persistent vulnerability after initial survival. This later mortality likely reflects a combination of injury severity, delayed complications, and limited critical care capacity, underscoring deficiencies across the full continuum of trauma care. In-hospital mortality patterns further demonstrate system-level constraints and triage underdevelopment. ICU deaths were markedly higher among critically injured patients (83.3%) compared with non-critical patients (16.7%), suggesting severe illness at ICU admission, while non-critical deaths might be due to potential delays in escalation. More than 71% of ward deaths occurred among critically injured patients, pointing to undertriage or insufficient ICU/HDU capacity.

Overall, similar to published studies [4, 5, 17, 18, 30–32], in this cohort, critically injured patients had more than twice the mortality of non-critical patients (17.6% vs. 8.2%), and mortality increased sharply with the number of critical criteria met, from 7.4% with one criterion to 75% with more than three. Hypotension at presentation carried a 25% risk of death, reinforcing its value as a key early severity marker. Deaths among patients initially classified as non-critical, including those later requiring ICU admission, likely reflect delayed recognition of deterioration and undertriage rather than failure of the severity classification, consistent with reports from other resource-constrained trauma systems [18, 23, 24]. Critically injured patients also had longer median hospital stays (8 days), while deaths occurred earlier (median 3 days), consistent with severe injury and limited critical care resources [33–35].

Survival analysis confirmed a strong relationship between early clinical severity, care setting, and outcomes. Nearly 39% of critically injured patients died within 14 days, compared with 2.8% of non-critical patients, demonstrating the discriminatory performance of the severity classification. Critically injured patients had more than twice the 14-day hazard of death (HR 2.21; *p* < 0.0001), validating the prognostic strength of the criteria.

Multivariable modeling identified critical injury as the strongest independent predictor of mortality (aOR 5.53; *p* < 0.0001). Higher mortality among transferred and rural patients highlights inequities in referral pathways, transport delays, and limited stabilization capacity [21–23, 36]. Increased mortality among ambulance arrivals likely reflects selective transport of severely injured patients and prolonged transport without formal emergency medical system support [37]. Delayed definitive care was associated with higher mortality (aOR 1.35; *p* = 0.04), reinforcing the need for faster access to definitive trauma interventions and stronger emergency care systems [9, 23, 25, 38–40]. A KTS ≤ 6 strongly predicted mortality (OR 3.78; *p* < 0.0001), supporting its utility as a pragmatic triage tool where advanced scoring is not feasible [20].

Sensitivity analyses further confirmed the robustness of the critical status classification despite minor data entry errors. The proportion of critically injured patients varied minimally (37%–39%), with consistently high mortality (17.6%–22.4%), reinforcing the stability and prognostic validity of the framework [4, 5, 17, 18, 41].

Taken together, the strong association between the number of critical criteria and mortality suggests that these physiological and injury-based triggers could be used to define a rapid trauma response in resource-limited settings. Because the criteria rely on basic clinical assessments available at first presentation, they may be implemented without advanced diagnostics or specialist staffing. This approach could enable standardized triage and early escalation of care, prioritizing high-risk patients for immediate interventions and higher-acuity monitoring when formal trauma teams are not available. It further highlights the importance of reliable trauma data systems to support real-time triage, monitoring, and surgical quality improvement in low-resource environments [14, 42, 43].

This study draws on a large, multicenter, prospectively collected inception cohort from Tanzanian referral hospitals, supported by one of sub-Saharan Africa’s few web-based trauma registries. The prospective design improves data completeness and temporal accuracy, reducing recall bias. A pragmatic physiology- and injury-based critical status definition supports scalable triage in resource-limited settings, while inclusion of both direct and transferred patients provides a comprehensive view of system-level care gaps. Adjusted logistics, survival, and sensitivity analyses strengthen internal validity. Limitations include pooled multicenter analyses without formal site-level effect estimation, which may obscure hospital-specific differences in resources and outcomes. Although hospital sites were included as a covariate, facility-level capacity data were inconsistently available, limiting comparative interpretation. Missing clinical details (e.g., neurologic data, imaging) reduces acuity precision, and ward versus ICU/HDU disposition may reflect capacity constraints rather than clinical need. Incomplete prehospital timestamps limit assessment of transfer delays, and the absence of provider- and hospital-level variables may introduce bias. Selection bias is possible as prehospital deaths were not captured, regression dilution may attenuate associations due to single-timepoint measurements, and exposure or outcome misclassification may arise from incomplete documentation. Categorization may still attenuate associations despite sensitivity analyses. Future studies should expand site-level data, integrate EMS timelines, and embed digital registries into routine care to support system-wide quality improvement.

## Conclusion

Trauma mortality remains high in this resource-limited setting, particularly among critically injured patients, many of whom are treated outside high-acuity care environments. Early physiologic derangements combined with high-risk injury features offer a feasible and scalable approach to identifying patients at greatest risk of death, enabling rapid triage where advanced diagnostics are unavailable. The graded association between the number of critical criteria and mortality supports using these triggers to guide early trauma response and escalation of care in low-resource systems. Strengthening early warning processes, expanding high-acuity capacity, and improving referral pathways are key opportunities to reduce preventable deaths. A web-based trauma registry is feasible and valuable for monitoring performance and informing system-level improvements. Future studies should validate these findings across additional centers and evaluate real-time triage and decision-support tools to optimize outcomes and advance trauma system strengthening goals.

## Supplementary Information

Below is the link to the electronic supplementary material.

Supplementary Material 1

## Data Availability

The data supporting the findings of this study are available from the corresponding author upon reasonable request.

## Abbreviations

AVPU: Alert, responds to Verbal stimuli, responds to Painful stimuli, Unresponsive
BP: Blood pressure
CI: Confidence interval
CT: Computed tomography
ED: Emergency department
HDU: High-dependency unit
HR: Hazard ratio
aOR: Adjusted odds ratio
ICU: Intensive care unit
IQR: Interquartile range
EMS: Emergency Medical Services
KTS: Kampala Trauma Score
LEF: Lower extremity fracture
LED: Lower extremity dislocation
LMICs: Low- and middle-income countries
MOI: Muhimbili Orthopedic Institute
MVC: Motor vehicle collision
OR: Odds ratio
SD: Standard deviation
SpO_2_: Peripheral oxygen saturation
STROBE: Strengthening the Reporting of Observational Studies in Epidemiology
UEF: Upper extremity fracture
UED: Upper extremity dislocation
SAS: Statistical Analysis System
