# Supplementary material for: Time-critical care gaps and systemic delays linked to higher mortality in severe trauma patients in Tanzania
Source: BMC Emerg Med. 2026 Feb 10;26:73. doi: 10.1186/s12873-026-01498-8 (PMC12990456; doi:10.1186/s12873-026-01498-8)
Supplement: Supplementary file 1 — Supplementary Material 1 [file 12873_2026_1498_MOESM1_ESM.docx]

**Supplemental File 1**

**List of Parameter Variables for sample size estimation, n=23**

Demographics and Background:

- Age
- Gender
- Occupation

Prehospital & Arrival Factors:

- Transfer Status
- Arrival Mode
- Mechanism of Injury
- Alcohol Use
- Trauma Origin (Urban vs. Rural)

Road Traffic Injury-Specific Data

- Vehicle (Type Of Vehicle Involved)
- Sector (Public, Private)
- Role_Road (Pedestrian, Driver, Passenger, Etc.)
- Helmet (Yes/No)
- Seatbelt (Yes/No)

Initial Clinical Presentation (Vital Signs & Severity Scores):

- Heart Rate
- Oxygen Saturation
- Respiratory Rate
- Blood Pressure (SBP/DBP)
- Conscious Level (AVPU)
- Injuries_Type (Head, Chest, Abdomen, etc.)
- Kampala Trauma Score (KTS)

Care & Treatment Factors:

- Discharged
- Admitted to Ward
- Admitted to ICU
- Taken to Operating Room (OR)
- Referred Out

Outcome Measures:

- Mortality at 24 hours
- Mortality at 14 days
- Survived (Discharged, Admitted to Ward, ICU, OR)
- Died (24 hours ED, 14 days)

Time-Critical Care Gaps (Delays in Care)

- Prehospital delay (Time From Injury To Hospital Arrival)
- Triage delay (Time From Arrival To Triage)
- Definitive care delay (Time from triage to definitive treatment)
